# Maximum likelihood estimators for colony-forming units

**DOI:** 10.1128/spectrum.03946-23

**Published:** 2024-07-23

**Authors:** K. Michael Martini, Satya Spandana Boddu, Ilya Nemenman, Nic M. Vega

**Affiliations:** 1 Department of Physics, Emory University, Atlanta, Georgia, USA; 2 Initiative in Theory and Modeling of Living Systems, Emory University, Atlanta, Georgia, USA; 3 Department of Biology, Emory University, Atlanta, Georgia, USA; Iowa State University, Ames, Iowa, USA

**Keywords:** CFU, colony count estimation, bacterial counts, MPN, dilution plating, maximum likelihood estimator, dilution experiments, most probable number

## Abstract

Measuring the abundance of microbes in a sample is a common procedure with a long history, but best practices are not well-conserved across microbiological ﬁelds. Serial dilution methods are commonly used to dilute bacterial cultures to produce countable numbers of colonies, and from these counts, to infer bacterial concentrations measured in colony-forming units (CFUs). The most common methods to generate data for CFU point estimates involve plating bacteria on (or in) a solid growth medium and counting their resulting colonies or counting the number of tubes at a given dilution that have growth. Traditionally, these types of data have been analyzed separately using different analytic methods. Here, we build a direct correspondence between these approaches, which allows one to extend the use of the most probable number method from the liquid tubes experiments, for which it was developed, to the growth plates by viewing colony-sized patches of a plate as equivalent to individual tubes. We also discuss how to combine measurements taken at different dilutions, and we review several ways of analyzing colony counts, including the Poisson and truncated Poisson methods. We test all point estimate methods computationally using simulated data. For all methods, we discuss their relevant error bounds, assumptions, strengths, and weaknesses. We provide an online calculator for these estimators.

Estimation of the number of microbes in a sample is an important problem with a long history. Yet common practices, such as combining results from different measurements, remain sub-optimal. We provide a comparison of methods for estimating abundance of microbes and detail a mapping between different methods, which allows to extend their range of applicability. This mapping enables higher precision estimates of colony-forming units (CFUs) using the same data already collected for traditional CFU estimation methods. Furthermore, we provide recommendations for how to combine measurements of colony counts taken across dilutions, correcting several misconceptions in the literature.

## INTRODUCTION

Extrapolation of viable microbial counts from suspensions of live cells is a longstanding—and surprisingly complicated—problem, which here we will explore computationally. The fundamental problem is simple: there exists a sample with some unknown concentration of live microorganisms, which an experimentalist wants to measure. For simplicity, we will assume that the sample in question was taken as, or has been resuspended into, some initial volume. That initial volume will be serially diluted (usually in a 10-fold series), and ﬁxed-volume aliquots (or sub-samples) of the resulting suspensions will be cultured. If these aliquots are plated in or on agar, the resulting data will be in the form of colony counts (colony-forming units, CFUs). Alternately, multiple aliquots may be taken from a single dilution and used to seed a number of wells or tubes of liquid culture, or a number of plates. Then, the number of volumes showing growth when seeded from a particular dilution, as a fraction of the total number of volumes inoculated, can be used to calculate the most probable number (MPN) of live agents in the initial volume (1, 2).

Best practice for this apparently simple and ubiquitous scenario has been the subject of debate for over a century (1, 3–16). CFU measurement is inherently subject to errors due to the stochastic nature of counts. This type of error is the primary concern in research settings, where microbes are grown in culture prior to quantiﬁcation. For reasons that are not clear, the substantial literature on microbial quantiﬁcation in food and environmental samples is not well known in research laboratories, and the practices and estimators used vary considerably from lab to lab.

Counting error is related to the density and number of colonies. At one extreme, when the sample is too concentrated, the number of resulting colonies will be too numerous to count (TNTC; sometimes “too many to count,” TMTC). At these high concentrations, colonies merge, breaking the assumption that each microbe corresponds to one colony (6, 17). At the other extreme, when the sample is very diluted, the number of colony initiating bacteria in the sample is subject to small-number statistical (sampling) ﬂuctuations, resulting in high relative error (ratio of the standard deviation to the mean) (18, 19). Crowding error is widely acknowledged, and TMTC colonies or plates are generally simply excluded from analysis [although there are uses for these data, see, for example, reference (20)]. However, understanding of small number errors is less common in wet-lab microbiology. Finally, experimental errors, such as inaccuracies in pipetting, can emerge and compound over the steps of a serial dilution. However, the latter source of error is expected to be negligible for equipment calibrated to usual standards, and technical replication further reduces effects of this variation (21).

Thus, the problem at hand is how can CFU density best be estimated from plate counts, given the error produced by sampling ﬂuctuations, colony crowding, and (to a lesser degree) pipetting? These errors will contribute differently to different experimental designs. For a single sample with initial volume *V*_0_ which is represented by one count of colonies *n*_*k*_ at one dilution *d*_*k*_ (because only one dilution was measured, or because only one spot or one plate in a series was countable), statistical error of counts [presumably but not necessarily (22, 23) Poisson] is inevitable, and pipetting error will contribute but may not be signiﬁcant. For a single sample represented by more than one count of colonies (representing counts at different dilutions within a single dilution series, and/or technical replicates where one sample was measured multiple times), the same errors apply, but pipetting bias may not be constant across measurements (for example, one failing O-ring on a multichannel pipette can lead to bias in a single column of a 96-well plate). If technical errors are reasonably small, these measurements should conform to expectations for Poisson sampling error (24).

It is critical to note that for multiple samples of the same type measured in parallel (including biological replicates), we can no longer expect variation across samples to reﬂect a Poisson-distributed sampling error. Variation across samples will be biological (or otherwise inherent) and demographic (accumulating over time) in addition to that due to sampling. This was the basis for the Nobel Prize-winning experiments of Luria and Delbrück, who used the distribution of ﬂuctuations to distinguish Darwinian vs Lamarckian evolution (25). This is also frequently the case in environmental samples, where different samples from the same source (e. g., water samples from different parts of the same lake) will produce measurements that have super-Poisson variation (aka, over-dispersed). In such cases, a substantial part of the variation is “real” due to inhomogeneities in the source (26) and counts may be better represented by a lognormal or negative binomial distribution (22, 23) than by the Poisson (26).

These considerations are substantial. Sample inhomogeneity is known to be a major source of variation between measurements in environmental and food samples (22, 27). Even apparently “well-mixed” samples, such as water samples or liquid cultures in a laboratory, can be very inhomogeneous especially if microbes have a tendency to clump (19). However, such considerations are case-speciﬁc, and dealing with these is beyond the scope of the present work. The larger problem of microbiological estimation from varied and inhomogeneous samples has been dealt with extensively elsewhere, for example, references (18, 22, 28).

Here, we will focus on point estimation of CFU density within an individual, homogeneous sample, which may be represented by a single set of measurements or by technical replicates, in which one sample is measured multiple times. The main objective of this paper is to propose methods for accurately estimating CFUs, while taking into account the effects of crowding and sampling ﬂuctuations, without losing valuable data from counts. Drawing from previous research (17–19, 29), we present simple analytical formulas that can be used to combine counts from different dilutions and to obtain precise CFU point estimates along with accurate error bars. First, we examine existing point estimate methods in the literature, assessing their strengths and weaknesses including pick-the-best, Poisson, and Poisson with a cutoff. We discuss the “Poisson with a cutoff” method, which clariﬁes the impact of crowding on CFU density estimation and demonstrates how to minimize the effects of sampling error by combining measurements of “uncrowded” counts. We explore the mathematical equivalence between different regimes of the common CFU estimators. Next, we introduce a crowding-explicit model to demonstrate the relationship between canonical plate-based counts and the most probable number method for presence/absence of growth in liquid media. This is achieved by considering colony-sized patches of the plate as equivalent to individual tubes. This extension of the domain of applicability of MPN to colony counts will potentially increase the accuracy of a whole class of experiments with no additional experimental costs. Finally, we computationally evaluate and compare all the point estimators we discussed for their bias and standard errors. If experimentalists require estimates beyond point estimation such as full distributions or need to include experimental uncertainties in the counting process, more detailed methods are available in the literature (19). However, if an experimentalist just requires a point estimate of the CFU density, we provide practical recommendations for experimentalists on how to select appropriate dilution and replication schemes and how to combine data from multiple observations. We also have provided a calculator for these estimators available on Hugging Face spaces, named CFUestimator (30).

## RESULTS

### A brief history of counts

CFUs are a proxy for the concentration of microbes within a sample. A standard experimental procedure for estimating CFUs consists of serially diluting homogeneous samples in a sterile aqueous buffer, then plating aliquots of these dilutions on growth-supporting agar and later counting the resulting colonies. If an appropriate dilution has been reached, each microbe will form an independent colony that is countable by eye. We assume for simplicity that all plates or tubes used for growth have the same ability to support growth of the organism(s) being studied, and that the sample is suﬃciently homogenized to ensure that microbes are free in solution and not adhered to one another or to a substrate (4). (In practice, these assumptions should be tested before choosing an estimator (18, 26); we discuss one such test in the next section.).

The simplest way of estimating CFUs is to multiply the number of colonies by the reciprocal of the dilution factor to ﬁnd the concentration of colony-forming microbes in the original suspension (1, 5, 29). For example, say, there is a single sample represented by one countable 10 cm plate in a dilution series, where we observe 100 distinct colonies after plating 100 µL of a 1:100 dilution (dilution 2 in a 10-fold series) from the original sample. In this case, following this simple procedure, we would obtain


(1) $$\frac{CFU}{Volume}=\frac{counts}{FracOriginalVolume}=\frac{100}{0.1mL\cdot 0.01}=100\cdot 10^{3}=10^{5}CFU/mL.$$

This is exactly equivalent to multiplying the number of counts by a volume correction factor [1/(size of aliquot in mL)] and multiplying by the base of the dilution series raised to the power of the number of dilution steps


(2) $$\frac{CFU}{Volume}=\frac{1}{0.1mL}\cdot 100colonies\cdot 10^{2}=100\cdot 10^{3}=10^{5}CFU/mL.$$

This simple calculation follows from a more general Poisson model, explained below. This method works reasonably well under ideal conditions: all samples should be represented by a single count of colonies, and each count should be large enough to minimize small-number sampling ﬂuctuations, and yet small enough to avoid crowding on the plate. When any of these conditions are not met, accurate estimation of CFU density becomes more complicated.

There is a broad literature of methods proposing to ensure that estimates of CFU density are “good.” A good estimator should be accurate. Formally, this means that such estimators should have the true value of the CFU density as their expected value. In other words, they must be unbiased. Good estimators must also be precise, so that variance in the estimate is small and samples are repeatable. Therefore, an ideal solution to this problem should provide an estimator that is provably unbiased and with a minimal variance. The solution to this problem is well known in statistics: if we can assume that data follow a speciﬁc probability distribution, then the maximum likelihood estimator (MLE) for that distribution will have these properties (18). While this is formally true only for very large samples, MLEs generally perform well even for relatively small samples. Furthermore, an ideal method should be straightforward to use in the hands of researchers without advanced mathematics background. Unfortunately, many of the available methods fail one or the other of these requirements, being either simple to use, but statistically sub-optimal, or mathematically correct, but incomprehensible to many bench scientists.

Straightforward-to-use methods focus largely on designing protocols that avoid data in error-prone extremes. For example, the FDA recommends (31) that the best dilution range for coliform bacteria results in 25 to 250 colonies per 10 cm spread plate, with the ideal count closest to 250. Restriction on the high end limits counting errors due to growth limitation by nutrient depletion as well as outright merging of colonies, which would bias the number of counts downward. Conversely, restriction on the lower end limits the sampling error associated with small numbers of counts. Speciﬁcally, under the assumption that counts represent random draws from a given sample and are, therefore, Poisson distributed, the error scales as the square root of the number of counts. Thus, for small counts, the error becomes an unacceptably large fraction of the mean. Within the example above, our dilution 2 count of 100 colonies should have a standard deviation (SD) of $\sqrt{100}=10$, giving a coefficient of variation (CV) of 10%. At dilution 3, we might obtain 10 counts, with an SD of $\sqrt{10}\approx 3.16$ and a CV of 31.6%.

From here, the simplest approach that is often used in practice is to choose only the plate or spot that has the “best” count in the acceptable range, and to estimate CFU density based on that single count. Often, only the dilution at the high end of the countable range is used since it has the smallest sampling ﬂuctuations; all other measurements are discarded (29). We call this the “pick-the-best” method for later reference. If counts in the acceptable range can be consistently achieved, this method is straightforward and reasonably accurate. However, discarding data is rarely advisable, and over- and under-crowded measurements can, in fact, be used to improve CFU estimates.

### Simplest “good” estimator: Poisson

One simple and reasonably accurate model for calculating CFUs assumes that the number of colonies are Poisson distributed, with variation due to sampling. That is, for a particular dilution, the mean colony count for that dilution is the same as the variance. By this model, the most likely estimator for the density of microbes is simply the ratio of the total number of colonies counted from all plates divided by the total amount of liquid used from the original sample in all plates (see Supplementary Information). If there is only one countable measurement for a given sample, this simpliﬁes to “pick-the-best.”

The Poisson model implicitly assumes that the original sample is well mixed and each microbe plated will result in its own separate and countable colony. It further assumes that experimental volume is spread uniformly across the agar surface, resulting in cells being randomly distributed, independent of the locations of where other cells landed. Formally, these assumptions mean that there is a uniform and well-mixed population density *r* of microbes per unit volume in an initial volume of liquid *V*. The liquid is diluted by a factor *d_k_* = *V_k_* / *V*, where *V_k_* is the volume of the liquid from the original sample used on the plate or the spot *k*. Plating will result in *n_k_* colonies, where *n_k_* is Poisson distributed with the parameter *λ* = *r d_k_V* = *rV_k_*. That is, the average number of colonies per experiment is *r d_k_V* with variance *r d_k_V*. Using these assumptions, the MLE of the density of microbes *r*_mle_ and its standard error are


(3) $$r_{\text{mle }}=\frac{\sum_{k}n_{k}}{V\sum_{k}d_{k}}=\frac{\sum_{k}n_{k}}{\sum_{k}V_{k}},\;\sigma =\frac{r_{\text{mle }}}{\sqrt{\sum_{k}n_{k}}}.$$

In other words, the best estimator for the concentration, *r*_mle_, is the total number of colonies divided by the total amount of the original volume of liquid used. However, as noted earlier, this ignores crowding and counting errors. In practice, this method should be avoided unless all measurements are from well-dispersed, uncrowded plates, as crowding effects can make a large difference in the estimator, resulting in under-estimating the microbial density as colonies merge and are under-counted.

If technical replicates exist (multiple measurements of the same sample; four or more such measurements are recommended), it is straightforward to test whether the data adhere to a Poisson distribution using the following test, known as the dispersion index test. If there are *j* measurements of a given sample, with average number of counts $\overset{-}{N}$ and a variance of counts $s_{N}^{2}$, then the index of dispersion *D*^2^ is


(4) $$D^{2}=\frac{\left(j-1\right)s_{N}^{2}}{\overset{-}{N}},$$

which will be distributed as χ^2^ with j − 1 degrees of freedom (18, 32). If *D*^2^ is greater than the upper 1 − *α* quantile of that distribution, where *α* is the needed signiﬁcant *P*-value, then we reject the null hypothesis that these replicates are drawn from the same Poisson distribution. This can indicate technical problems that are introducing an excess (or insuﬃciency) of variation, possibly by biasing replicates differently from one another (e.g., the failing O-ring example above), or biases due to a too lenient cutoff for countability.

### Combining data: common bad estimators

The primary reason for the “pick-the-best” approach is that it eliminates confusion over how to combine multiple measurements for a given sample, particularly when counts belong to more than one dilution. First, notice that combining measurements from technical replicates that are taken at the same dilution is straightforward. For example, let us assume an original 200 µL volume *V* contains *r* = 3 · 10^8^ CFU. We can create simulated serial dilutions from this original volume by assuming that each pipetting step (10-fold dilutions and plating onto agar) is a binomial sampling event (19) that comes with experimental noise. In one such simulation, triplicate plating 100 µL aliquots results in counts *n*_6_ = (162, 141, 148), all from the sixth 10-fold dilution. The fraction of the original volume plated in each case is *V*_6_ = 0.5 ⋅ 10^−6^ = 5 ⋅ 10^−7^. These numbers can be combined via the Poisson method shown in the previous section to estimate CFU density in *V*,


(5) $$CFU=\frac{162+141+148}{5\cdot 10^{-7}+5\cdot 10^{-7}+5\cdot 10^{-7}}=\frac{162+141+148}{3\cdot 5\cdot 10^{-7}}=3.007\cdot 10^{8}.$$

Alternately, counts taken from the same dilution can be averaged across technical replicates, then adjusted by the volume plated and the dilution read (33)


(6) $$CFU=2\cdot \frac{162+141+148}{3}\cdot 10^{6}=2\cdot \frac{162+141+148}{3\cdot 10^{-6}}=\frac{162+141+148}{3\cdot 0.5\cdot 10^{-6}}=2.007\cdot 10^{8}.$$

Clearly, these two most common approaches are algebraically identical.

In contrast, combining counts across different dilutions is less straightforward. In fact, some commonly used methods for combining measurements are statistically inadmissible. For example, if there are multiple measurements in the countable range, the USDA FSIS recommends (33) that researchers calculate the estimated CFU for each dilution separately using the average colony count across technical replicates at a given dilution and then average the results of the separate dilutions. If the two estimates are more than a factor of 2 apart, the researcher is instructed to instead only use the counts from the higher-density plates. This commonly used method incorrectly combines the data using a simple average, thus increasing the variance of the estimated CFU density. Indeed, continuing the example above, let us suppose that, on the plates corresponding to the seventh 10-fold dilution from these three technical replicates, we observe (18, 22, 25) colonies. The Poisson estimator gives us


(7) $$\mathrm{CFU}=\frac{162+141+148+13+17+20}{3\left(5\cdot 10^{-7}\right)+3\left(5\cdot 10^{-8}\right)}=3.036\cdot 10^{8}.$$

The USDA averaging method gives


(8) $$CFU=\frac{\;1}{2}\left(2\cdot \frac{162+141+148}{3}\cdot 10^{6}+2\frac{13+17+20}{3}\cdot 10^{7}\right)\;\;\;\;\;\;\;\;\;\;\;\;\;\;\;\;\;\;\;\;\;\;\;\;\;\;\;=\frac{1}{2}\left(3.06\cdot 10^{8}+3.33\cdot 10^{8}\right)=3.2\cdot 10^{8}.$$

On these data, averaging was substantially less precise, with an error of 7% as compared with the Poisson method’s error of 1% (recall that the true density in this example is 3.0 · 10^8^ CFU per 200 µL). The USDA method improperly averages across dilutions, amplifying ﬂuctuations associated with small colony number counts, whereas the simple Poisson model properly combines measurements across dilutions by effectively re-weighting small counts by the small volumes with which they are associated. In a later section, we demonstrate that averaging across dilutions will, as a rule, increase the variance of CFU estimates.

### Too few and too many

Furthermore, there is the problem of what to do with zero counts. These data are inevitably limited by some threshold of detection (TOD), representing the smallest CFU density at which counts can be detected. This “left-censoring” is a well-known issue (34–36) with many proposed work-arounds, including but not limited to substituting zeros with a small value (which may be the average of the undetectable range, a maximum likelihood inferred value, or some other small number), reporting zeros as “below TOD” or “<1” rather than as a value, and pretending they did not happen (not generally recommended; although if zeros are rare, it will not make much difference) (34, 36). Sometimes, a threshold of quantiﬁcation representing the lowest “acceptable” (suﬃciently precise) count is used along with or instead of TOD (29), with values below this threshold omitted from analysis.

The “best” approach to zero-contaminated count data depends on what else is in the data and what the data will be used to do. We assume here that the goal is to obtain a point estimate of CFU density in the original sample (as opposed to, for example, determining the probability that this density is in excess of some threshold). If a sample is represented by zero and non-zero measurements, the Poisson model explicitly allows zero counts to be incorporated as outcomes of the random sampling process. For example, if a hypothetical *V* = 200 µL sample contains 5 · 10^7^ CFU, one simulation of serial dilution and plating in triplicate with 100 µL per plate produces dilution 6 counts of (31, 26, 20 ) and dilution 7 counts of (4, 0, 0). Using just the dilution 6 counts, we estimate


(9) $$CFU=\frac{31+26+20}{3(5\cdot 10^{-7})}=5.13\cdot 10^{7}\pm 0.59\cdot 10^{7}.$$

If we use the lower dilution as well, we obtain


(10) $$CFU=\frac{31+26+20+4+0+0}{3\left(5\cdot 10^{-7}\right)+3\left(5\cdot 10^{-8}\right)}=4.91\cdot 10^{7}\pm 0.55\cdot 10^{7}.$$

In this case, incorporating data from zeros (in the form of additional volume that was plated but contained no counts) improved precision. Alternately, when zeros are common because the density in the original sample is close to the TOD, non-zero counts are useful for making a distinction between samples where no organisms are detectable (and density might be zero) and those where the density of organisms cannot be zero. Although the actual density cannot be estimated accurately or precisely from very low counts, the distinction between “<TOD” and “>1” for a given sample is important (36).

At the other end of the range, researchers must deal with crowding and set thresholds for “too many to count.” Deﬁning an optimal range for “countable” data is not always straightforward, and this determination is very important to ensure that CFU estimates are accurate. Since the sampling-based standard error of counts scales as $\frac{1}{\sqrt{\sum_{k}n_{k}}}$, the number of colonies counted *n*_*k*_ should be as large as reasonably possible.

However, there are consequences for pushing this too far. As cell density in the aliquot increases, counts will be biased downward due to merging of colonies and colony stunting or failure to grow. These data are then “right-censored,” with an upper limit past which the number of counts observed does not increase in proportion to an increase in the density in the original sample. Densities above this point result in “crowded” samples, with counts that are lower than the true number of CFUs. Furthermore, as the number of colonies per plate or spot increases, data collection becomes more time-consuming or requires expensive robots for automatic counting; it is common for researchers to minimize effort on plates near the top of the “acceptable” range by dividing plates into sections, counting colonies in one section, and multiplying this count by the number of sections to get an estimated ﬁnal count for the whole plate. While this approach is sufficient for a rough estimate of CFU density, it introduces additional sampling variation due to both reduction in counts and imperfect division of plates, and it does not remove bias due to crowding. We will demonstrate the consequences later in this paper.

Previous works (17) have modeled crowding using shifted Poisson distributions. In these models, the variance of estimates from crowded data would be the same as if there was no crowding and the mean would be shifted down due to colonies merging together. However, this is *a priori* unlikely to be true. As we will show below, if colonies are crowded, both the mean and the variance will be shifted relative to the pure Poisson (uncrowded) distribution. The reason for this is that the variance of the large colony counts is shifted downward due to a “ceiling” effect—there is an upper bound to the total number of colonies, which limits upward ﬂuctuations. In other words, the use of a shifted Poisson distribution is a reasonable approximation, but the variance must also be modiﬁed.

### Better estimators: Poisson with cutoff, aka what is countable, exactly?

The main problem with the I Poisson model is that it does not account for counting errors due to crowding. The simplest way to take account of the crowding is to assume that there is a threshold of colonies, *M*, below which crowding is negligible, which in practice will often be smaller than the largest number of counts we are willing to attempt (TMTC). It is convenient to assume that this threshold *M* is the same as some TMTC threshold (e.g., the commonly referenced 250–300 colonies per plate for coliforms). However, this threshold will depend on colony size and morphology, which will vary across different bacteria and will change for a given bacterial strain depending on the media used, concentration of agar in a given batch of plates, incubation time and temperature, humidity, etc. To be certain of the validity of a chosen threshold within a standardized protocol, it is advisable to plate a ﬁner-than-normal dilution series (two- to ﬁvefold, to ensure multiple dilutions with readable counts) and check that dispersion within and across different “countable” dilutions for a given sample is consistent with Poisson. For densities where crowding affects counts, the data will become “right-censored” and show a decrease in variation as compared with expectations for Poisson-distributed samples.

Once the threshold *M* is determined, we can then segment our data into two parts: plates/spots with counts above the threshold where crowding is important, and plates/spots with counts below this threshold for which crowding is not important. If we have identiﬁed our cutoffs well, for our theoretical homogeneous sample, the Poisson estimator is correct for all measurements *k* where the number of colonies counted *n_k_* ≤ *M*. The calculation is, therefore, exactly the same as for the I Poisson estimator, with the caveat that only measurements *n_k_* ≤ *M* are used. Here, the indicator function *I* (*n_k_ < M*) is 1 when *n_k_ < M*, and 0 otherwise. Similarly, *I* (*n_k_ > M*) is 1 when *n_k_ > M*, and 0 otherwise. Due to its balance between simplicity and accuracy, this method is the easiest to use in practice:


(11) $$r_{\text{mle }}=\frac{\sum_{k}I\left(n_{k}\leq M\right)n_{k}}{\sum_{k}I\left(n_{k}\leq M\right)V_{k}},\;\sigma =\frac{r_{mle}}{\sqrt{\sum_{k}I\left(n_{k}\leq M\right)n_{k}}}.$$

If we want to incorporate data from measurements above this threshold *M*, the calculation becomes slightly more complicated. This method was ﬁrst introduced in the literature as “averaging TNTC counts” (18, 20). Using “crowded” measurements as if they were uncrowded will bias the naive Poisson estimator downward, resulting in under-estimation of CFU density (Fig. 2). However, we can use the number of plates/spots that were above the crowding threshold *M*, along with the colony counts from plates/spots below this threshold at the same dilution, to estimate CFUs. This will be applicable when plate counts at a given dilution are toward the high end of the countable range, such that some technical replicates fall below this threshold and others above it by chance. To estimate the CFU density in the original sample *r*, the following equation should be solved numerically (see SI for the derivation):


(12) $$\sum_{k}I\left(n_{k}\leq N\right)\left(\frac{n_{k}}{r}-d_{k}V\right)+\sum_{k}I\left(n_{k}>N\right)\;d_{k}\;\frac{d_{k}V{\left(d_{k}rV\right)}^{N}e^{-d_{k}rV}}{\int_{0}^{d_{k}rV}t^{N}e^{-t}dt}\;=0.$$

The ﬁrst term is equivalent to the simple Poisson model and uses the counts from uncrowded samples directly, whereas the second term reﬂects the probability of counts being above the threshold *M*. Inference of *r* can be done in Excel using SOLVER or using numerical solvers in R, Python, MATLAB, etc. An equivalent model is shown in references (18, 20).

This model properly accounts for two error sources in counts from an individual sample: the sampling ﬂuctuations and the crowding effect. The simple Poisson, using only counts from uncrowded plates, gives a good estimate for the CFU counts and properly combines multiple measurements at different dilution factors. The more sophisticated form of the model has greater precision, but the greater computational effort may or may not be worth it to an investigator depending on the effect size and the structure of the experiment. In the next section, we present an alternate estimator based on the most probable number approach, which we argue provides a better tradeoff between effort and estimator performance when incorporating data from crowded samples.

### Crowding and the most probable number

For the ﬁnal model, we consider the effects of crowding in space. To account for crowding, we will divide each plate into *N* ≈ $\frac{A_{plate}}{A_{colony}}$ regions, each approximately the size of a full colony. We make the assumptions that if more than one microbe lands in one of these regions, the colonies that form from these cells will grow together and be counted as one colony. For each region, the number of cells landing in that region will be Poisson distributed with parameter $\lambda =\frac{d_{k}rV}{N}$.

These assumptions are equivalent to that of quantal-based methods for microbial quantiﬁcation, such as the commonly used MPN method. In the MPN assay, a known quantity (volume of original sample) is introduced into each of a series of *N* replicate tubes, and the dilution of the original sample is adjusted to ﬁnd a region where some of the tubes contain viable growth and some do not. The results of this assay are therefore, for each dilution volume *V_k_* from the original sample, out of the *N_k_* tubes inoculated, a number *n_k_* that is positive for growth.

**Fig 1:**
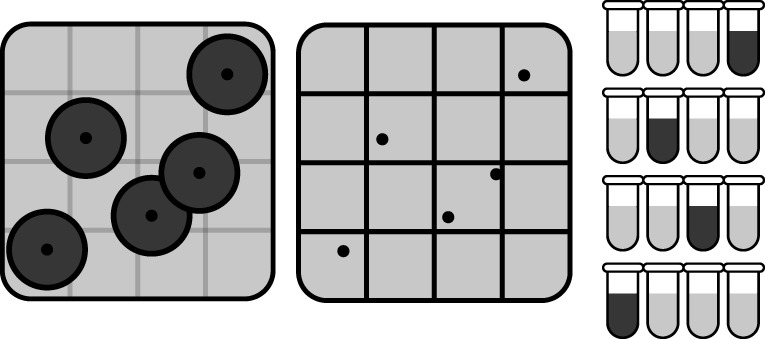
Visual equivalence between plate and tube-based assays. The left panel is a cartoon of a typical plate containing colonies, where the growing colonies are shown as dark disks. In the middle panel, the plate is divided into *N* (here 16) approximately colony-sized regions. If a region contains one or more colony centers (black dots), this region can be mapped to a positive (dark) tube as shown in the right panel. Similarly, regions containing no colony centers are mapped to negative (light) tubes. This demonstrates that plating is equivalent to a massive parallel version of a tube-based assay with $N\approx \frac{A_{plate}}{A_{colony}}.$ Furthermore, it demonstrates that the MPN method can be used for plate data.

A direct mapping to tube-based assays is possible if space on a plate (or within a spot) is considered as a set of colony-sized bins. Each of the *N* colony-sized regions on a plate or within a spot corresponds to one tube. The presence of colonies in a particular region corresponds to when a tube has growth. Hence, a plate that is divided into *N* regions can be thought of as *N* tubes being tested in parallel, cf. Fig. 1.

Therefore, the probability of *n_k_* successes in *N* colony-sized regions on the agar surface can be described using a crowding-explicit model based on the binomial distribution. Assuming that the cells in the original sample are well mixed, the probability of zero cells landing in a particular region is (from the Poisson) $p_{0}=e^{-d_{k}rV/N}$ and the probability that at least one cell lands in that region is therefore $p_{§amp;gt;}=1-e^{-d_{k}rV/N}$. Assuming that the original sample is well mixed, each region is independent of all other regions in our crowding model, so that


(13) p(nk)=(Nnk)p>nkp0N−nk=(Nnk)(1−e−dkrVN)nke−dkrV(N−nk).

We can maximize this probability to ﬁnd the MLE CFU density, *r*_mle_ (see the SI for the full derivation). We can accomplish this by numerically solving the following equation for *r*:


(14) $$\sum_{k}\frac{d_{k}n_{k}}{N\left(1-e^{-rd_{k}V/N}\right)}=\sum_{k}d_{k}.$$

This expression for *r* is the same as that of the MPN estimator (31, 37, 38). In the SI, we show that, in the limit where concentrations and colony counts are low, this model simpliﬁes to the Poisson model. Outside the “uncrowded” regime, the mean and the variance of data from the crowding model are not the same as in the Poisson. Therefore, the two approaches are not equal to each other, though both are depressed due to the “ceiling” effect described earlier. In the SI, we also ﬁnd that the error associated with the maximum likelihood estimator *r*_mle_ of the MPN method can be minimized at an optimal dilution factor, which falls into the crowded regime.

The MPN procedure can generate biased estimates of the original sample density, and the precision and accuracy of results depend strongly on the number of tubes used (18). The bias on the maximum likelihood estimator results in an over-estimate of 20%–25% with five tubes, which is reduced to a few percent with 50 tubes (see SI). By back of the envelope calculation, an average 10 cm plate (inside diameter 86 mm, surface area 58 cm^2^) can ﬁt a maximum of approximately 5,000 medium-sized (1 mm outside diameter) “tubes,” whereas a single grid square on a 10 × 10 cm square plate (typically gridded 6 × 6) can ﬁt 200 of these colony-sized spaces. All of these are well above the threshold where the bias in this estimator (39) makes much difference in the value. (Note that this refers to the number of colony-sized spaces available and is independent of the number of colonies observed.) This also means that the standard error of the estimator will, in theory, be minimized at a plating density that is much higher than the threshold for “uncrowded” plates and, in fact, is well into a range of densities where a minority of colonies will be distinct. Fortunately, the standard error is still well behaved over a broad space in fraction of regions occupied (SI), meaning that plate counts into the “uncrowded” range will still produce good estimates with this method. In fact, this produces a result equivalent to that of the Poisson method in the fully uncrowded regime. However, the MPN method is most useful as plating densities encroach into the crowded regime, allowing precise and accurate estimation of CFU density from plates that would provide severely biased estimates using a naive Poisson model.

### Utility of the models

Here, we demonstrate the relative utility of each model for estimation of CFU density from simulated data. First, we can use the crowding-explicit binomial sampling model described in the previous section to estimate bias due to crowding, and to demonstrate the importance of choosing an appropriate cutoff *M*, below which plates are considered to be uncrowded and countable. To do so, we solve the crowded binomial model in equation 13 for *dV* with respect to the average number of colonies ⟨*n*⟩ and the number of colony-sized regions on a plate *N*. Doing so, we ﬁnd $dV=-\frac{N}{r}log(1-\frac{\langle n\rangle }{N})$. We can substitute this into the Poisson estimator and find


(15) $$r_{p}=\frac{\left\langle n\right\rangle }{dV}=\frac{\left\langle n\right\rangle }{-\frac{N}{r}ln\left(1-\frac{\left\langle n\right\rangle }{N}\right)}=-r\frac{\frac{\left\langle n\right\rangle }{N}}{ln\left(1-\frac{\left\langle n\right\rangle }{N}\right)}.$$

Let us define the ratio of expected colony number to the number of colony-sized regions as $f=\frac{\langle n\rangle }{N}$. This ratio represents the amount of crowding, where a value of 1 is the maximum crowding and a value close to zero is in the uncrowded regime. Expressing the previous expression in terms of the crowding, we see


(16) $$\frac{r_{p}}{r}=-\frac{f}{\ln\left(1-f\right)}.$$

**Fig 2:**
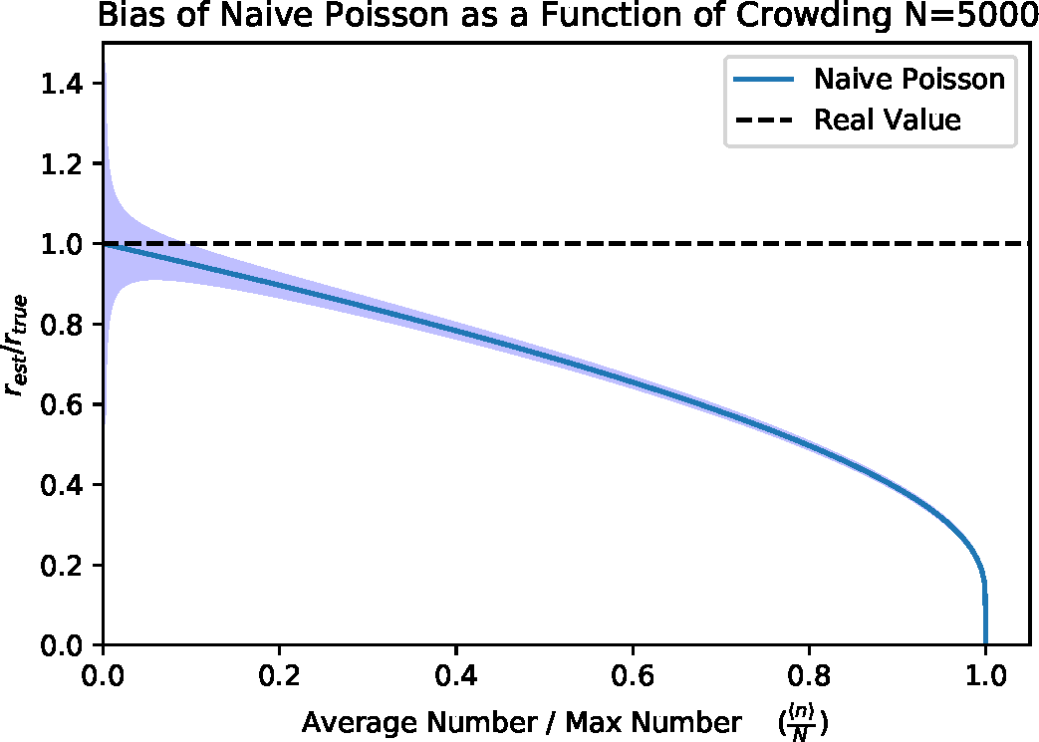
Bias and under-estimation of the true bacterial concentration as a function of crowding, illustrated using the Poisson estimator. We illustrate this by plotting the ratio of the estimated concentration (with the error bands denoting ±1 SEM at *N* = 5,000) to the true concentration. Here, crowding is measured by the ratio of the average number of colonies to the maximum number of colonies that can ﬁt within a plate $f=\frac{\left\langle n\right\rangle }{N}$. At low crowding values (encompassing the conventionally recommended 25–250 colonies per plate), the naive estimator has low bias, but large uncertainty. At a crowding value of 0.2 (∼1,000 colonies on a 10 cm plate—an ambitious task, and not recommended), the naive Poisson estimator under-estimates the true concentration by about 10%, and many-fold under-estimation is possible as crowding approaches 1.

This ratio indicates how close the estimated CFU concentration is to the true concentration. A ratio of 1 tells us that we have an unbiased estimator, whereas a ratio of less than 1 tells us we are under-estimating the CFU density. We plot this expression in Fig. 2 to show how the simple Poisson estimator under-estimates the actual concentration as a function of crowding, *f*. After a crowding value of *f* = 0.2, the Poisson estimator starts to be signiﬁcantly biased, undershooting the true value by about 10%. This has implications for the value used in the Poisson model with a cutoff. The cutoff should be chosen such that the bias is not greater than the experimenters targeted precision. For example, if a bias must be less than 10%, then a cutoff of about 20% of the total plate capacity should be used. In the case of a 10 cm plate with an estimated 5,000 1 mm diameter colony-sized regions, this corresponds to a cutoff of *M* = 1,000, whereas the more typical cutoff of *M* = 300 provides an essentially unbiased estimate (bias 3%), but this results in a large statistical ﬂuctuation of 5.8%. In the case of a 6 by 6 mm grid on a 10 cm by 10 cm plate, there are roughly 200 grid regions in a plate. Thus, an *M* = 40 would be appropriate to achieve bias less than 10%, and a threshold of *M* = 12 colonies is required to reduce bias to 3% for colonies of this size. At these thresholds, the statistical error would be 15.8%.

**Fig 3:**
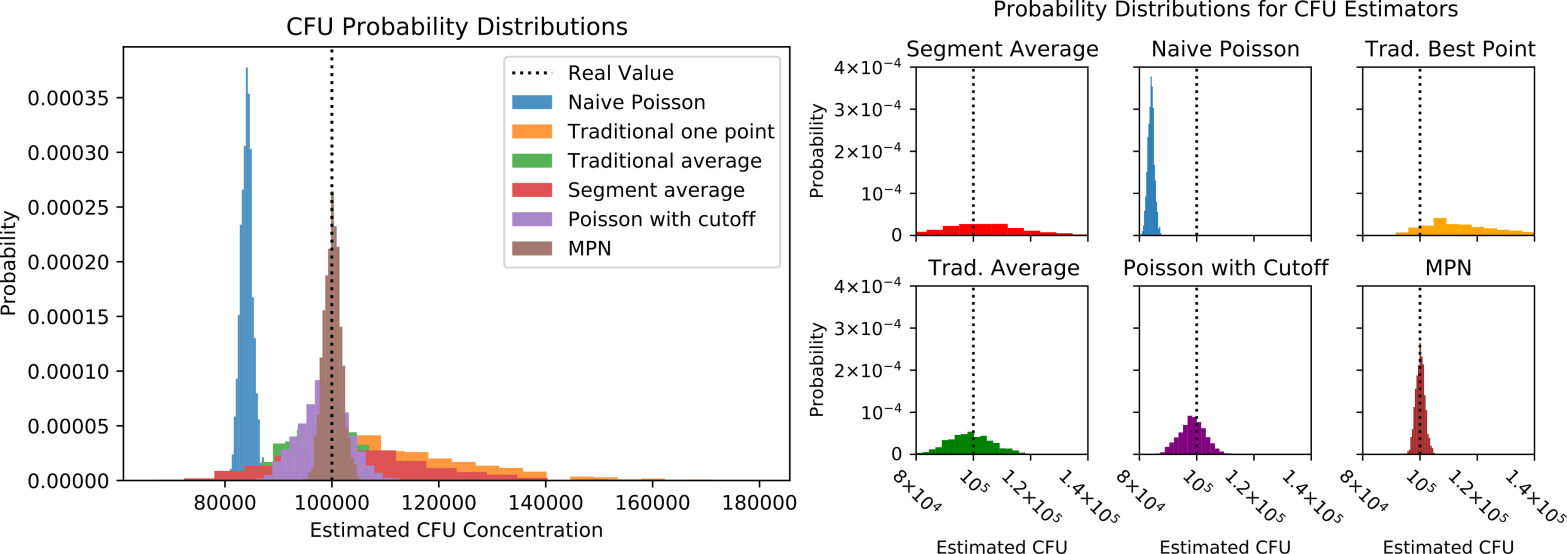
The probability distributions of estimated CFU concentrations from different estimators generated from 1,000 independent numerical experiments with dilutions 0.1, 0.1, 0.01, 0.01, 0.001, 0.001, *r* = 100,000, *V* = 0.2, *N* = 5,000. Here, the segmented-plate average (one-quarter of the plate is counted), naive Poisson, “pick the best,” traditional average, Poisson with cutoff, and MPN methods are compared. The MPN method demonstrates the best combination of high precision and accuracy.

To compare the performance of the different estimators discussed here, we simulated 1,000 experiments and applied each of our estimators to the resulting data. Data for each experiment was modeled using the binomial crowding model with *r* = 10^5^, *V* = 0.2, *N* = 5,000, and dilution values (0.1, 0.1, 0.01, 0.01, 0.001, 0.001). This corresponds to two replicates for each dilution in a 10-fold dilution experiment. An example set of colony counts corresponding to these dilutions is (1,705, 1,629, 196, 181, 21, 21). The ﬁrst two dilutions are in the over-crowded regime and the last two dilutions are in the dilute uncrowded regime. The traditional methods (“pick-the-best,” averaging, segment averaging) and Poisson with a cutoff will discard the ﬁrst two counts as too many to count, while the other methods will use their numeric values. The resulting distributions are plotted in Fig. 3.

The results show that the MPN method is unbiased and has the highest degree of accuracy. The Poisson with a cutoff (which always discards counts from the least-diluted samples in these outputs) is nearly unbiased, whereas the naive Poisson (which unrealistically uses all colony counts and included here only for comparison) is biased down due to inclusion of “crowded” data. This illustrates the relative costs of discarding data and using these data inappropriately. The naive Poisson has a similar variance as that of the MPN because both are using all the data points. However, the measure around which the naive Poisson estimator varies is incorrect due to this bias. With the Poisson estimator, increasing accuracy comes at a cost in precision; the Poisson with cutoff has roughly twice the standard error of the MPN method due to the fact that it does not use all the data and throws out the ﬁrst two counts of each experiment. Next, the traditional averaging method (33) has roughly ﬁve times the standard error of the MPN method, due to the fact that it gives lower-precision measurements the same weight as higher-precision large counts in the uncrowded regime. However, it is unbiased. If there are technical replicates, pick-the-best (choosing the largest number of counts in the countable range, over multiple technical replicates at each dilution) is a biased estimator (over-estimating CFUs) and has a standard error roughly 10 times that of the MPN method. Pick-the-best where the best count from each technical replicate is used is equivalent to Poisson with a cutoff, with some loss of precision due to discarding of small counts. Segment averaging (here, counting one-quarter of the plate, and assuming perfect segmenting such that exactly one-quarter of the colonies are counted) resulted in an unbiased estimator with the largest standard error, roughly 13 times the standard error of the MPN method.

These simulations show that the MPN method produces the most precise results and is unbiased. However, the Poisson with a cutoff is a close second, also with high accuracy and precision and with the advantage of being practical to calculate by hand. The bias of the naive Poisson (using all data) serves as a warning: if counts are not in the uncrowded regime, the Poisson assumptions do not apply, and an estimator using only number of colonies counted at each dilution will under-estimate the CFU density in the original sample. Other standard estimators (averaging, segment averaging) using the same data required for the Poisson estimator show universally poorer precision than Poisson with a cutoff and cannot be recommended.

## DISCUSSION

We have discussed commonly used point estimator methods for estimating CFUs in a single sample from dilution series data, and we have presented a new method based on the MPN framework. The methods overview given here, while far from comprehensive (19, 40–44), is intended as a practical introduction to sampling errors in count-based measurements, particularly for researchers outside environmental and food microbiological surveillance who are likely to have had little if any exposure to existing literature.

We have focused here on technical rationales for choosing a point estimator for CFU density, but it is important to emphasize that the research question and the data taken must be suitable. We assume here that the researcher wishes to obtain a point estimate of bacterial density in each sample, with high accuracy and precision so that comparisons between samples can be made. The Poisson-based estimator used throughout this paper assumes well-mixed, homogeneous samples with high culturability, which may not be true even for shaken broth cultures. This assumption should be veriﬁed for the sample type to be used. Departures from the Poisson will appear as deviations from expected dispersal of counts (18, 23), as indicated in the text. If measurements across technical replicates are not Poisson, this may be correctable. For example, if bacteria are known or suspected to clump, proper shaking (6) and/or use of a surfactant like Tween 80 (45, 46) can help to disperse aggregates. As always, any such protocols should be validated under the conditions where they are to be used.

In practice, the choice of point estimator will depend on the precision required for the estimate of CFU density, as well as the tradeoffs between experimental repetition and analytical complexity that a researcher is willing to make. We have provided a summary of the strengths and weaknesses of all methods in Table 1. Traditional pick-the-best estimators are ﬁne for quick imprecise measurements; however, this method has the largest standard error as it does not use a majority of the data collected. Other methods can use more of data collected to provide more precise point estimates of the concentration with smaller standard error. For experiments with reasonably large expected effect size, the simplest mathematically admissible method—the Poisson estimator with a cutoff—is perfectly valid, as long as the dilutions are chosen appropriately to ensure all measurements are in the countable range. Broadly speaking, addition of unbiased data will improve the precision of an estimator. Historically, technical replicates have been used for this purpose—even technical duplication is sufficient to markedly reduce variance of the estimated CFU density, although triplicate plating is preferred to safeguard against accidents and outliers (28) (also see SI). The Poisson model allows data from technical replicates to be combined into a single mathematically interpretable point estimator with deﬁnable properties—speciﬁcally, a maximum likelihood estimator, which should be an unbiased and minimally variable estimator for the true value. This is as opposed to averaging (33), which produces an estimate whose properties are not well deﬁned. The Poisson method also allows the investigator to incorporate data from dilutions with too few counts, in addition to (not in place of) data from countable wells in the same dilution series—by effectively re-weighting the contribution of these wells by the total volume of original suspension that they contain, these data can be used to improve the accuracy of the estimator even though their sampling variance is high. We have presented several methods for estimating CFUs and we have provided a calculator for these estimators available on Hugging Face spaces, named CFUestimator (30).

**TABLE 1:** Table summarizing the strengths and weaknesses of each estimator along with their appropriate regions of validity

Estimator name	Strengths and weaknesses
Pick the best	This traditional estimator is simple to understand and calculate but has large standard error.
Naive Poisson	The naive Poisson is valid at low crowding, but if used with high crowding data can produce a strong bias. It can combine data across multiple different dilutions.
Poisson with cutoff	The Poisson with a cutoff is easy to calculate by hand. It can combine data from different dilutions. The resulting bias of the estimator can be controlled by setting the cutoff and is suﬃciently small when data are uncrowded.
MPN	The MPN method is valid across all crowding levels and uses all available data. It can combine multiple experiments. The same method can be used for colony counts on plates and in tubes, viewing patches of a plate as equivalent to individual tubes. The method requires either a computer program or table to calculate. It produces an unbiased estimator and has the smallest standard error of all discussed methods.

The correspondence shown here between using tubes and gridding a plate into subsections based on colony area allows the usage of estimator techniques typically used for quantal-based measurements of CFU density, speciﬁcally the MPN, where positive growth events (e.g., colonies) are explicitly considered to represent one or more originating cells. These techniques have a long history in environmental surveillance microbiology, and statistically well-founded techniques are readily available for analysis of such data (2, 47, 48). If an experimentalist wants tighter bounds for an estimated CFU count, the MPN provides a very low-variance, unbiased estimator at the cost of some extra steps. This estimator allows the experimentalist to incorporate data from normally uncountable (TMTC) plates as well as counts from uncrowded plates, maximizing the amount of information that can be gleaned from a dilution series.

The MPN model requires an estimate of the maximum number of colonies that can be packed into the growth area for each sample; we show (SI) that it is better to over-estimate this maximum than to under-estimate it. If the patch size on a plate is correctly chosen to be around the size of a typical colony, even a spot-plating assay on a 10 by 10 cm plate is equivalent to running hundreds of tubes in parallel. Furthermore, it is necessary to estimate the number of occupied regions in the growth area. In or near the uncrowded regime, this will be equivalent to the number of counts. However, this method does not require that all colonies are individually countable—instead, image analysis (49–51) can be used to estimate both the size of an individual colony and the fraction of total area occupied by colony growth. The MPN estimator can therefore potentially provide accurate, precise estimates of CFU density for plates where exact counts cannot be obtained. However, colony size varies across different microorganisms as well as across culture conditions (media type, agar percentage, pad thickness, plate drying time and conditions, growth temperature and atmosphere, etc.) and incubation time on plates, meaning that the size range of colonies may be different even across plates within a single experiment (52, 53). This added complication of properly choosing a grid size or determining the typical size of a colony means that application of the MPN will most likely require parameters estimated for the speciﬁc experiment being analyzed. Furthermore, the fact that colony size can decrease under crowding means that heavily crowded plates or plate regions, where few or no distinct colonies are visible, may have very different “average” colony sizes than the same microbes in a less-crowded area. While theory suggests that the MPN estimator will be most precise when the majority of colony-sized locations are occupied [(54), also see SI], this practical limitation suggests that use of the MPN on plate count data will become less accurate with extremes of crowding, and that the best use of the MPN is likely to be in the liminal region between the technically uncrowded and the physically uncountable, where most to all growth is in the form of distinct, countable colonies but crowding produces a measurable bias in these counts.

## MATERIALS AND METHODS

Simulations were coded in Python. We provided a custom calculator for all estimators analyzed in this work on Hugging Face spaces, named CFUestimator (30).

## SUPPLEMENTAL MATERIAL

Supplemental material spectrum.03946-23-s0001.pdf Maximum likelihood estimators for CFU concentration.

ASM does not own the copyrights to Supplemental Material that may be linked to, or accessed through, an article. The authors have granted ASM a non-exclusive, world-wide license to publish the Supplemental Material files. Please contact the corresponding author directly for reuse.
