# Supplementary material for: Maximum likelihood estimators for colony-forming units
Source: Microbiol Spectr. 2024 Jul 23;12(9):e03946-23. doi: 10.1128/spectrum.03946-23 (PMC11371269; doi:10.1128/spectrum.03946-23)
Supplement: Supplemental material — Maximum likelihood estimators for CFU concentration. [file spectrum.03946-23-s0001.pdf]

Supplementary Material for Maximum Likelihood Estimators For Colony Forming Units

K. Michael Martini **Satya Spandana Boddu** **Nic Vega** **Ilya Nemenman**

1 Introduction

In this Supplementary Material, we mathematically derive the Maximum Likelihood Estimators for CFU concentration presented in the Main Text. Section 2 reviews the basic Poisson model for CFU estimation, Section 3 derives the Poisson model with a cutoff, and Section 4 derives the MPN method applied to crowded plate data. Table 1 defines a dictionary of variable names and symbols, and Table 2 presents a summary of the estimators discussed in the Main Text. Finally, Section 6, discusses the bias of the naive Poisson estimator for crowded data.

We briefly summarize the problem and the relevant variables as follows: In a typical experiment, there is a stock liquid with an unknown concentration of microbes r. This initial liquid is divided between multiple plates or tubes and can be diluted by different factors. In the case of plates, this is with the goal of producing low enough concentrations, such that any resulting colonies can then easily be counted. Similarly, in tubes, the aim is to have multiple tubes, such that some contain viable stock, and some do not. Each plate and/or tube contains the same volume of liquid V. However, the concentration has been diluted by a factor $d_k = V_k/V$, where V_k is the amount of liquid from the original sample used in the dilution for the kth tube or plate. This implies that, for an experiment k, we have a volume V_k of liquid with density $d_k r$ cells per unit volume. Colonies are allowed to grow on each plate, and the total number of colonies is counted as n_k.

All the following methods assume that the microbes are randomly distributed throughout each sample, that microbes do not cluster or attract one another, that their growth is independent of one another, and that the medium is not selective.

2 Poisson Model

The Poisson model of population counts of microbes assumes that there is a uniform population density r of microbes per unit volume in an initial volume V_0 of liquid. The liquid is well mixed and will result in n_k colonies, where n_k is Poisson distributed with a parameter $\lambda = rd_kV$. That is, the average number of colonies per experiment is rd_kV with variance rd_kV. Each experimental volume is spread uniformly across entire plates resulting in cells being randomly distributed across the plate. The locations of the initial cells are uniformly random and are independent of the locations of where other cells landed. Additionally, it is assumed that each microbe will grow up into a full colony, which then can be counted independently of all other colonies. The Poisson model assumes no crowding.

The likelihood that an experiment k, with dilution rate d_k, has n_k colonies is

$$P(n_k) = \frac{(d_k rV)^{n_k} e^{-d_k rV}}{n_k!}. \quad (1)$$

The combined likelihood of all experiments is

$$L = \prod_k P(n_k) = \prod_k \frac{(d_k rV)^{n_k} e^{-d_k rV}}{n_k!}. \quad (2)$$

The value of concentration, r, that maximizes this likelihood or equivalently the value of r that maximizes the log likelihood, is known as the maximum likelihood estimator (r_{mle}). The maximum likelihood estimator of concentration is an unbiased estimator given the data follows the same probability distribution as given by the model. In reality, the data follows a different distribution.

To find r_{mle}, we find the maximum of the log-likelihood by setting its derivative w. r. t. r to zero:

$$\frac{\partial \ln(L)}{\partial r} = \frac{\partial}{\partial r}\left(\sum_k [n_k \ln(d_k r V) - d_k r V - \ln(n_k!)]\right) = \sum_k \left[\frac{n_k}{r} - d_k V\right] = 0. \tag{3}$$

This implies

$$r_{\text{mle}} = \frac{\sum_k n_k}{V \sum_k d_k} = \frac{\sum_k n_k}{\sum_k V_k}. \tag{4}$$

The standard error of the maximum likelihood estimator can be calculated from the second order derivative of the log-likelihood w. r. t. r:

$$\frac{-1}{\sigma^2} = \frac{\partial^2}{\partial r^2}\ln(L) = \frac{-\sum_k n_k}{r^2} \tag{5}$$

After some algebra, the variance of the mle becomes

$$\sigma^2 = \frac{r_{\text{mle}}^2}{\sum_k n_k} = \frac{r_{\text{mle}}^2}{K\langle n\rangle} = \frac{\sum_k n_k}{(V\sum_k d_k)^2}. \tag{6}$$

3 Poisson Model with a Cutoff

In the Poisson model with a cutoff, it is assumed that colonies are still distributed via a Poisson distribution. However, above a critical colony count, M, the colonies start to merge and the experimenter will not count the colonies, instead assigning the category of "too crowded to count". The probability that colony counts are above the threshold M is calculated as

$$1 - \sum_{i=0}^{M} \frac{(d_k r V)^i e^{-d_k r V}}{i!} = \frac{\gamma(M+1, d_k r V)}{M!} = \frac{\int_0^{d_k r V} t^M e^{-t} dt}{M!}, \tag{7}$$

where $\gamma(N+1, d_k r V)$ is the lower incomplete gamma function. We use indicator functions to write the likelihood of colony counts, where the indicator function $I(n < M)$ is 1 when $n < M$, and 0 otherwise. Similarly, $I(n > M)$ is 1 when $n > M$,and 0 otherwise. For the purpose of writing the likelihood, if the number of colonies n_k is greater than the threshold M, we will assign it the count of $M + 1$ which will act as a special category corresponding to all plates that are too crowded to count. The likelihood is then:

$$L = \prod_k P(n_k) = \prod_k \left[\frac{(d_k r V)^{n_k} e^{-d_k r V}}{n_k!}\right]^{I(n_k \le M)} \left[\frac{\int_0^{d_k r V} t^M e^{-t} dt}{M!}\right]^{I(n_k > M)}. \tag{8}$$

We find the value of r that maximizes the the log likelihood in a manner similar to the previous section:

$$\begin{aligned}\frac{\partial \ln L}{\partial r} &= \frac{\partial}{\partial r}\sum_k \Bigg[I(n_k \le M)(n_k \ln(d_k r V) - d_k r V - \ln(n_k!)) \\ &+ I(n_k > M)\left(\ln \int_0^{d_k r V} t^M e^{-t} dt - \ln(M!)\right)\Bigg] \\ &= \sum_k I(n_k \le M)(\frac{n_k}{r} - d_k V) + \sum_k I(n_k > M)\frac{d_k V (d_k r V)^M e^{-d_k r V}}{\int_0^{d_k r V} t^M e^{-t} dt} = 0. \end{aligned} \tag{9}$$

This expression can be solved for numerically for r.

There are several limits of this equation that can be easily understood. First, in the uncrowded dilution regime, where $d_k r V \ll 1$, the mle estimator of r becomes

$$r_{\text{mle}} = \frac{\sum_k [I(n_k \leq M)n_k + (M+1)I(n_k > M)]}{\sum_k V_k}. \tag{10}$$

A more useful limit is when there are no data points where $d_k r V \approx M$ and the data falls into two categories, either $d_k r V \ll M$ or $d_k r V \gg M$. In this limit, the the second term in equation 9 corresponding to colony counts larger than M can be ignored. This is equivalent to the normal Poisson solution, but with all colony counts larger than the maximum M being ignored. The resulting solution is:

$$r_{\text{mle}} = \frac{\sum_k I(n_k \leq M)n_k}{\sum_k I(n_k \leq M)V_k}. \tag{11}$$

In general, this simple approximate formula gives a result almost identical to the exact numerical solution for r in this regime.

4 Binomial Model of Crowding (MPN)

To account for crowding,we will divide the plate into N regions each the size of a full colony. We then make the assumption that if more than one bacterium lands in one of these regions, the colonies that would form from these cells will grow together and be counted as one colony. For each region, the number of cells landing in that region will be Poisson distributed with parameter $\lambda = \frac{d_k r V}{N}$. The probability of 0 cells landing in a region is $p_0 = e^{\frac{-d_k r V}{N}}$, and the probability of more than one cell landing in a region is $p_> = (1 - e^{\frac{-d_k r V}{N}})$. The number of colonies observed will be binomial distributed

$$p(n_k) = \binom{N}{n_k} p_>^{n_k} p_0^{N-n_k} = \binom{N}{n_k} (1 - e^{\frac{-d_k r V}{N}})^{n_k} e^{-d_k r V (N-n_k)}. \tag{12}$$

Again, we can find the r that maximizes the log-likelihood for this crowding model.

$$\begin{aligned} \frac{\partial \ln L}{\partial r} &= \frac{\partial}{\partial r} \sum_k \left[\ln \left(\binom{N}{n_k} \right) + \frac{(-r d_k V)(N - n_k) + n_k \ln(1 - e^{-r d_k V/N})}{N} \right] \\ &= \sum_k \left[\frac{-d_k V}{N}(n - n_k) + \frac{n_k e^{-r d_k V/N}}{1 - e^{-r d_k V/N}} \right] \\ &= \sum_k \left[\frac{d_k n_k V}{N(1 - e^{-r d_k V/N})} - d_k V \right] = 0. \end{aligned} \tag{13}$$

We can numerically solve the last equation to find r. One limit that is exactly solvable is when $d_k = d$. If we had K trials where all the dilutions where exactly the same, the best estimate for r becomes $r_{\text{mle}} = -\frac{N}{dV} \ln(1 - \frac{\sum_k n_k}{KN}) = -\frac{N}{dV} \ln(1 - \frac{\langle n \rangle}{N})$. This is actually directly related to the expected number of colonies found from the binomial distribution, which is $\langle n \rangle = N(1 - e^{-rdV/N})$. Similarly, the binomial distribution will have a variance of the expected number of colonies of $N(1 - e^{-rdV/N})(e^{-rdV/N})$.

In the small concentration limit $rdV/N \ll 1$, the mean and the variance are approximately the same, rdV, which would be the case for the Poisson distribution. This shows that the crowding model reduces to the Poisson model for low concentrations and low colony counts. However, if you keep to the next highest order in our small parameter, we see that the mean and variance are actually different from one another, namely $\langle n \rangle \approx rdV - \frac{r^2 d^2 V^2}{2N}$ and $\text{Var}(n) = rdV - \frac{3r^2 d^2 V^2}{2N}$.

Previous work [1] has modeled crowding using the shifted Poisson distributions. They claimed the variance would be the same as if there was no crowding, and the mean would be shifted down due to colonies merging together. However, this is inconsistent with our model of crowding. In fact, both the mean and the variance are shifted relative to what their true values would have been if the distributions were purely Poisson. The reason that the variance of large colony counts is also shifted downwards is that we have an upper bound on the total number of colonies, above which the estimator cannot fluctuate since the colonies would merge and be counted as single colonies. In other words,

the use of a shifted Poisson distribution is a fine approximation, but the variance—and not just the mean—must also be adjusted.

We can find the error associated with the maximum likelihood estimator for r as before, by finding the second order derivative of the log likelihood with respect to r:

$$\begin{aligned} \frac{\partial^2 \ln L}{\partial r^2} &= \frac{\partial}{\partial r} \sum_k \left[\frac{d_k n_k V}{N(1-e^{-r d_k V/N})} - d_k V \right] &(14)\\ &= -\sum_k \frac{d_k^2 V^2 n_k e^{-r d_k V/N}}{N^2(1-e^{-r d_k V/N})^2} &(15)\\ &= \frac{-1}{\sigma_r^2}. &(16) \end{aligned}$$

Switching again to the case where we are conducting K trials at the same dilution $d_k = d$, we find $\sigma_r^2 \approx \frac{\langle n \rangle}{d^2 V^2 K (1+\langle n \rangle/N)} \approx \frac{N(e^{rdV/N}-1)}{d^2 V^2 K}$. We can attempt now to find if there is an optimal dilution rate that would give us the smallest uncertainty. For this, let $u = \frac{rdV}{N}$. Then the variance becomes $\frac{r^2(e^u-1)}{Ku^2}$. The function $\frac{(e^u-1)}{Ku^2}$ has a minimum when $u \approx 1.594$, which implies the optimal dilution $d_{\text{opt}} = 1.594N/(rV)$. Alternatively, the error is minimized when the average number of colonies is roughly 80% of the maximum total number of colonies possible.

We estimate the size of a region for applying the MPN estimator to plates as the typical size of a colony, so that the maximum number of regions in a plate, N, is the ratio of the plate area to the typical colony size area. To understand how sensitive MPN is to this choice, we simulated data from the binomial crowding model with fixed $N_{\text{true}} = 5000$ and applied the MPN estimator to the simulated data, while varying the maximum estimated number of colonies N. We generated data for $r = 100000$, $V = 0.2$, $N = 5000$, and dilution values d_k=0.1, 0.1, 0.01, 0.01, 0.001, 0.001. Figure 1 shows the functional dependence of the estimated value on the guess for N. This plot illustrates that the estimator reduces to the naive-Poisson estimator as the max number of colonies approaches infinity. It also shows that, if the measured number of colonies from an experiment is close to the guessed N, the estimator diverges. Indeed, there are many concentrations that correspond to a fully crowded plate. This analysis shows that it is better to use an overestimate for the max number of colonies than an underestimate. An overestimate will, at worst, result in the estimate given by the Naive Poisson estimator. An underestimate, on the other hand, will result in greatly overestimating the role that crowding plays and will bias the estimate of the concentration upwards.

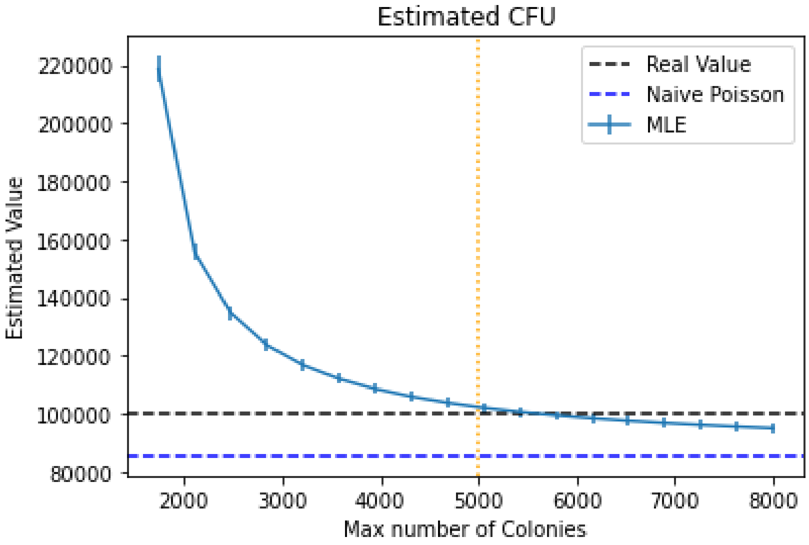

Figure 1: The MPN estimator as a function of an estimated maximum number of colonies, N, when the true maximum number is 5000.

5 Summary of Methods

Table 1 summarizes variable names, their corresponding symbols, and any relevant notes about the variables. Table 2 summarizes how to calculate different estimators and the corresponding standard error for each estimator.

Variable Name	Symbol	Notes
CFU concentration	r	The concentration of microbes (counts per volume)
total volume of sample	V	The total volume of a sample
volume of original sample	V_k	The volume of the original sample used for sample k
number of counts	n_k	The number of colony counts for sample k
dilution factor	d_k	The dilution factor $\frac{V_k}{V_0}$ sample k
cutoff threshold	M	The threshold of colonies, above which there are crowding effects
maximum number of colonies	N	The maximum number of colony-sized regions in a plate.<br>The maximum colony count that can fit on a plate $N \approx \frac{A_{\text{plate}}}{A_{\text{colony}}}$

Table 1: A table summarizing variable names and meanings

Estimator Name	Estimator	Standard Error
Naive Poisson	$\frac{\sum_k n_k}{\sum_k V_k}$	$\sqrt{\frac{r_{\text{mle}}^2}{\sum_k n_k}}$
Poisson with cutoff	$\frac{\sum_k I(n_k \le M) n_k}{\sum_k I(n_k \le M) V_k}$	$\sqrt{\frac{r_{\text{mle}}^2}{\sum_k I(n_k \le M) n_k}}$
Sophisticated Poisson with cutoff	$\sum_k I(n_k \le M)(\frac{n_k}{r} - d_k V) \quad +$<br>$\sum_k I(n_k > M) \frac{d_k V (d_k r V)^M e^{-d_k r V}}{\int_0^{d_k r V} t^M e^{-t} dt} = 0$	$\sqrt{\frac{r_{\text{mle}}^2}{\sum_k I(n_k \le M) n_k}}$
MPN	$\sum_k \frac{d_k n_k}{N(1-e^{-r d_k V/N})} = \sum_k d_k$	$\sqrt{\frac{\langle n \rangle}{d^2 V^2 K (1+\langle n \rangle/N)}}$

Table 2: A table summarizing the estimator methods along with their corresponding standard errors.

6 Bias of the Poisson estimator applied to crowded data

We use the above binomial model of crowding to get an estimate of the bias of the simple Poisson model applied to crowded data collected at the same dilution $d_k = d$. In this situation, the Poisson estimator is $r_p = \frac{\sum_k n_k}{KdV} = \frac{\langle n \rangle}{dV}$. We substitute into this expression the expected number of colonies from the binomial crowding model to find how well the Poisson estimator works under these conditions.

$$r_p = \frac{\langle n \rangle}{dV} = \frac{N(1 - e^{-rdV/N})}{dV}. \tag{17}$$

Similarly we find how the standard error should behave as a function of dilution.

$$\sigma^2 = \frac{r_p^2}{K\langle n \rangle} = \frac{r_p^2}{KN(1 - e^{-rdV/N})}. \tag{18}$$

We simulated data from the binomial crowding model and applied the Poisson estimator to the data generated from the model. We generated data for $r = 100000$, $V = 0.2$, $N = 5000$, and d ranging from 10^{-4} to 10^{-1}. The following Figs. 2, 3, 4 show the theoretical curves along with the predictions for specific data generated at the given dilutions and different number of replicate measurements. Notice how very low dilutions (corresponding to a few counts per plate) give unbiased but higher-variance estimates. Also note that, up to a dilution of about $5 \cdot 10^{-2}$, both estimators give relatively unbiased results. However, for dilution factors approaching $5 \cdot 10^{-2}$ (corresponding to about 1000 colonies per plate), the Poisson estimator starts to give very biased estimates undershooting the true value of the concentration.

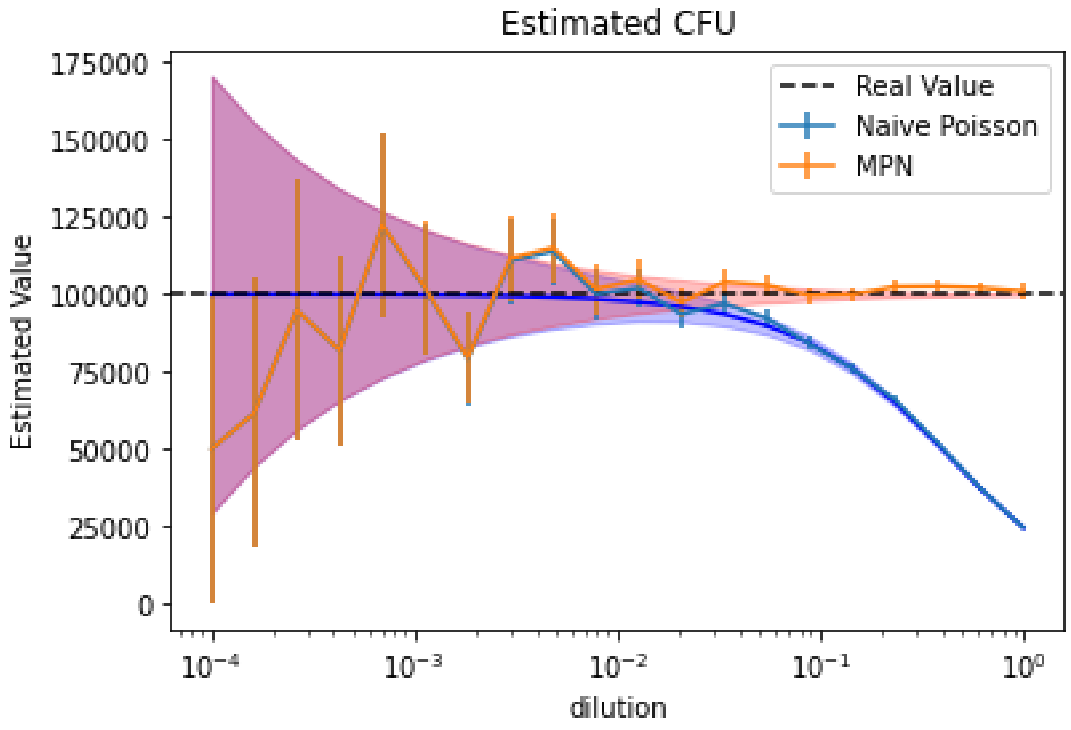

Figure 2: The naive Poisson estimator and the MPN estimator for one realization per dilution of data drawn from a binomial crowding model. Shaded regions correspond to the theoretical standard error of both estimators.

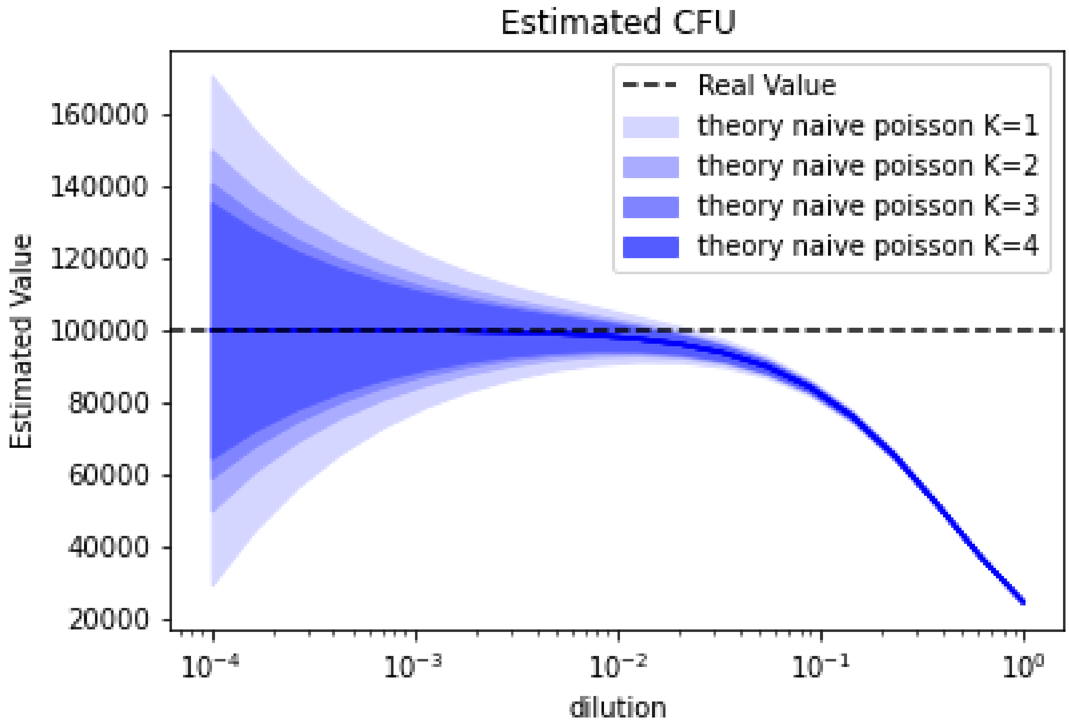

Figure 3: The naive Poisson estimator with standard error for replicates $K = 1$, $K = 2$, $K = 3$, and $K = 4$.

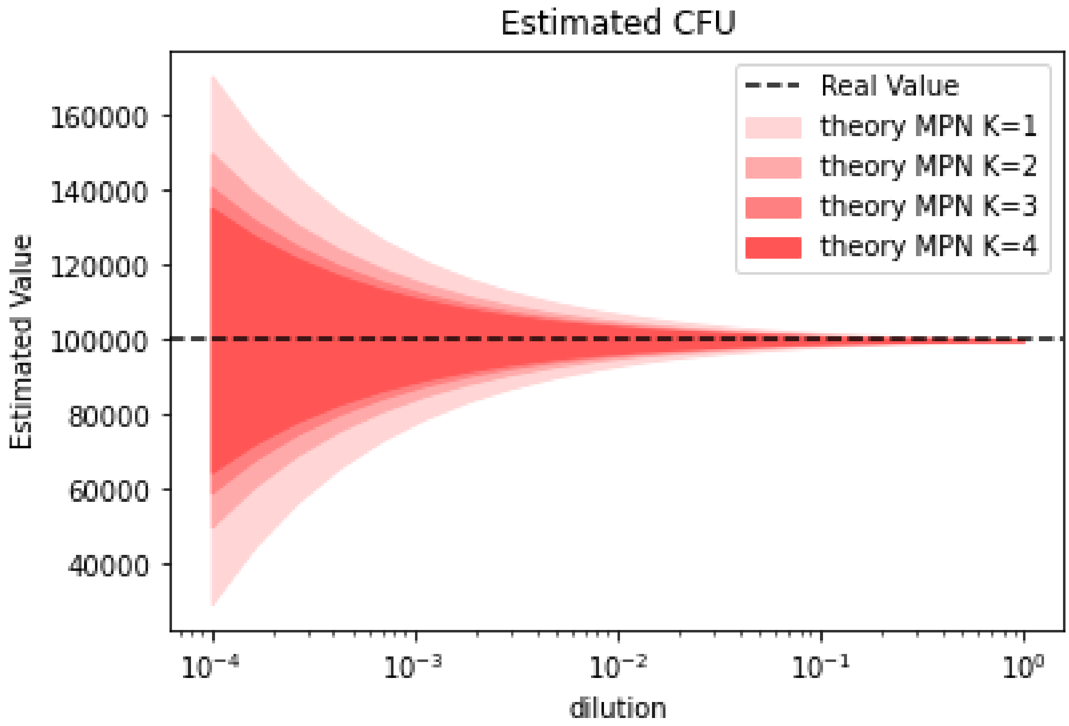

Figure 4: The MPN estimator with standard error for replicates $K = 1$, $K = 2$, $K = 3$, and $K = 4$.

Another way to view this data is to solve the crowded binomial model for dV with respect to the average number of colonies and maximum number of colonies allowed. Doing so we find $dV = \frac{-N}{r}\ln(1-\frac{\langle n\rangle}{N})$. We can substitute this into the Poisson estimator and find:

$$r_p = \frac{\langle n\rangle}{dV} = \frac{\langle n\rangle}{\frac{-N}{r}\ln(1-\frac{\langle n\rangle}{N})} = -r\frac{\frac{\langle n\rangle}{N}}{\ln(1-\frac{\langle n\rangle}{N})}. \tag{19}$$

Let us define the ratio of the expected colony number to the maximum colony number as $f = \frac{\langle n\rangle}{N}$. This ratio represents the amount of crowding, a value of 1 is the maximum crowding and a value close to zero is in the uncrowded regime. Expressing the previous expression in terms of the crowding, we obtain:

$$\frac{r_p}{r} = -\frac{f}{\ln(1-f)} \tag{20}$$

This ratio indicates how close the estimated concentration is to the true concentration. A ratio of 1 tells us that we have an unbiased estimator, a ratio of less than 1 tells that we are underestimating the true value of the concentration. We plot this expression in Fig. 2 in the Main Text to show how the Poisson estimator underestimates the actual concentration as a function of crowding, f. After a crowding value of $f = 0.2$ the naive Poisson estimator starts to be significantly biased, undershooting the true value by about 10%. This has implications for the value used in the Poisson model with a cutoff. The cutoff should chosen such that the bias is not greater than the experimenters targeted precision. For an error less than 10%, a cutoff of about 20% of the maximum number of colonies should be used. In the case of a maximum of 5000 colonies, this corresponds to a cutoff of $M = 1000$.

References

[1] Avishai Ben-David and Charles E Davidson. Estimation method for serial dilution experiments. *Journal of microbiological methods*, 107:214–221, 2014.
